# The use of indocyanine green and technetium-99 for dual-tracer sentinel lymph node biopsy in breast cancer

**DOI:** 10.1007/s10549-025-07767-7

**Published:** 2025-06-27

**Authors:** Madison Kolbow, Qianyun Luo, Alicia Cerrato Grande, Schelomo Marmor, Jennifer Witt, Sydne Muratore, Todd M. Tuttle, Jane Y. C. Hui

**Affiliations:** 1 University of Minnesota Medical School, Minneapolis, MN USA; 2 Breast Department, Division of Surgical Oncology, La Liga Contra el Cáncer, San Pedro Sula, Honduras; 3 Division of Surgical Oncology, Department of Surgery, University of Minnesota, Minneapolis, MN USA; 4 Core for Quality Outcomes, Discovery and Evaluation, University of Minnesota, Minneapolis, MN USA; 5 Department of Surgery, Minnesota Oncology, Minneapolis, MN USA; 6 Department of Surgery, Intermountain Health, Peaks Region, Denver, CO USA

**Keywords:** Sentinel lymph node mapping, Indocyanine green, Sentinel lymph node biopsy, Breast cancer surgery

## Abstract

**Purpose:**

The aim of this study was to determine if indocyanine green (ICG) is a suitable replacement for blue dye for dual-tracer sentinel lymph node biopsy (SLNB).

**Methods:**

A single-center retrospective review of female breast cancer patients aged ≥ 18 years who underwent SLNB with technetium-99 (Tc^99^) and ICG was performed from November 2022 to April 2024. Operative reports were reviewed to determine sentinel lymph node (SLN) identification rates with ICG (fluorescent) and Tc^99^ (radioactive). Pathology reports were reviewed to determine the pathology of excised SLNs.

**Results:**

One hundred and nineteen SLNBs were performed on 117 patients. At least one radioactive or fluorescent SLN was identified in 93.2% of all patients. The mean number of SLNs retrieved per SLNB was 1.6 (fluorescent, 1.5; radioactive, 1.5). Of all excised SLNs, 89.4% were fluorescent, 88.4% were radioactive, and 81.9% were both fluorescent and radioactive. SLN metastases were present in 26 patients (22.2%); of SLNs identified with metastases on pathologic examination, 87.2% were fluorescent, 74.4% were radioactive, and 71.8% were both radioactive and fluorescent. Two patients (1.7%) experienced skin flap necrosis and one patient (0.9%) experienced prolonged skin discoloration. No patients experienced allergic reactions.

**Conclusion:**

This study demonstrates that SLN identification rates using ICG and Tc^99^ are comparable to those using blue dye and Tc^99^. Thus, ICG is a suitable alternative for blue dye. Future work should assess if ICG is a suitable tracer for SLNB in low-resource settings where Tc^99^ is not available.

## Introduction

Dual-tracer sentinel lymph node biopsy (SLNB) with technetium-99 m sulfur colloid (Tc^99^) and blue dye is the current standard of care for staging of the axilla for patients with early stage breast cancer. Blue dye is an umbrella term for tracers such as methylene blue and isosulfan blue, both of which have been associated with perioperative complications. These complications range from mild hypersensitivity reactions to anaphylaxis, skin tattooing, and skin and fat necrosis [1–3]. Compared with other tracers, blue dye requires additional downtime after injection prior to SLNB. Breast massage for up to five minutes is recommended for maximal dilation of lymphatic channels and adequate tracer travel to sentinel lymph nodes (SLNs) [4]. Given these drawbacks, indocyanine green (ICG) has been explored as an alternative to blue dye in SLNB in recent years.

In contrast to blue dye, ICG is thought to avoid adverse effects associated with blue dyes. ICG uniquely provides rapid visualization of lymphatic channels and SLNs after injection during SLNB [5]. Importantly, ICG has been found to be a reliable tracer for the detection of SLNB as a standalone tracer or in combination with blue dye or Tc^99^ [6–17]. The objective of this study was to determine if ICG is a suitable replacement for blue dye in dual-tracer SLNB.

## Methods

**Fig. 1:**
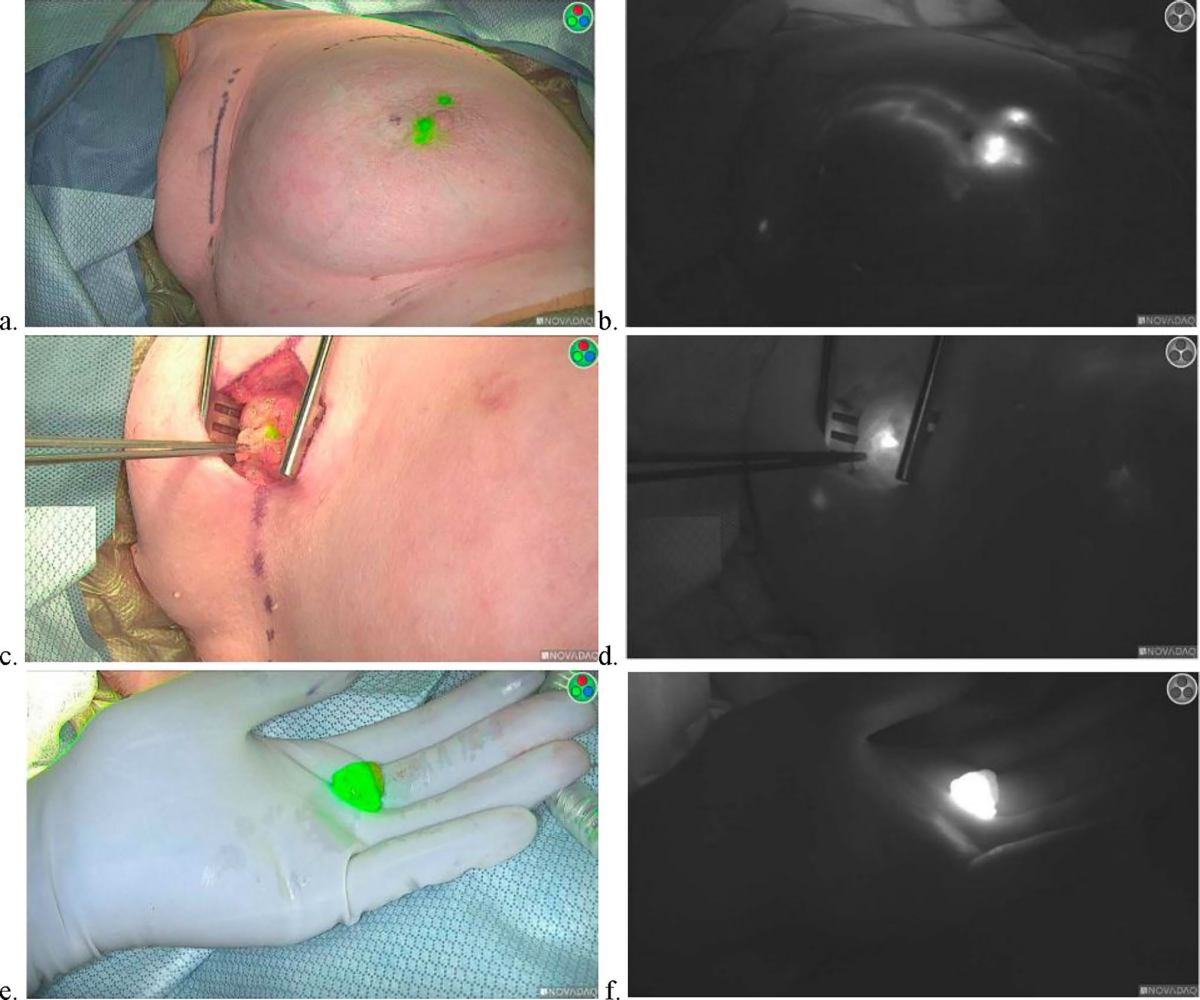
Sentinel lymph node mapping after injection of indocyanine green, shown in near infrared fluorescence with overlay (**a**) and without (**b**). The mapped sentinel lymph node in vivo is visualized in situ with overlay (**c**) and without (**d**), and then is excised, shown with overlay (**e**) and without (**f**)

After University of Minnesota Institutional Review Board approval, a retrospective chart review was conducted to evaluate the suitability of ICG as a replacement for blue dye for dual-tracer SLNB. Female breast cancer patients ≥ 18 years of age who underwent SLNB with ICG and Tc^99^ within a single academic health system from November 2022 to April 2024 were reviewed. Male patients and patients who did not consent to participate in research were excluded. The authors affirm that the human research participant provided informed consent for the publication of Fig. 1.

### Mapping technique

ICG mapping was performed by injecting a total of 0.1 ml of ICG intradermally into the areolar skin of the right breast at the 12 and 9 o’clock positions or of the left breast at 12 and 3 o’clock positions preoperatively, just prior to skin preparation and draping the patient in the operating room. Post-injection massage was not performed. Intraoperatively, the SPY Portable Handheld Imager was utilized to visualize the fluorescent injection sites, lymphatic channels, and SLNs (Fig. 1). For breast-conserving surgery, whether the breast surgery or the axillary surgery was performed first was at the discretion of the operating surgeon. Tc^99^ mapping was performed by injecting Tc^99^ into the retroareolar breast preoperatively. Intraoperatively, a gamma detector was utilized to identify SLNs with Tc^99^ uptake.

### Data

Two surgeons maintained a registry of patients who had undergone SLNB with ICG and Tc^99^ in a Health Insurance Portability and Accountability Act (HIPAA) compliant server. Operative reports were reviewed to determine sentinel lymph node detection with ICG and Tc^99^, receipt of breast-conserving surgery or mastectomy, whether or not patients underwent immediate reconstruction, and if there were any immediate complications. Pathology reports were reviewed to determine the pathology of excised SLN. Patient charts were manually reviewed to determine basic demographic information, smoking status, tumor and treatment characteristics, and postoperative complications.

Rural–urban status, insurance type, and area deprivation index (ADI) were provided by the Clinical Quality, Outcomes, Discovery, and Evaluation (CQODE) Core. The ADI is a measure of socioeconomic disadvantage at the neighborhood level, developed based on factors such as income, education, employment, and housing quality [18]. The ADI data used in this study were derived from publicly available census data and standardized to national rankings to allow for comparisons across geographic regions. These data were collected and curated by CQODE to ensure accuracy and consistency.

### Data analysis

Multivariable logistic regression was performed to determine the factors associated with sentinel lymph node detection by indocyanine green. Analyses were performed with R, Version 4.3.0 (R Foundation for Statistical Computing; Vienna, Austria). A two tailed p value of ≤ 0.05 was selected to reflect statistical significance.

## Results

### Study population characteristics

Throughout the study period, 117 patients underwent a total of 119 SLNBs. The median age at the time of SLNB was 59 years. The median BMI of the study population was 26.9 [15.3 to 53.1]. Most of the patients were White (84%) with the rest of the study population identifying as Black/African American (3%) or having other or unknown race (13%). The majority of the population were nonsmokers (87%) (Table 1). Other characteristics are found in Table 1.

**Table 1:** Patient characteristics

	*n* (%)
*N*	117
Median age at surgery, years	59
Median BMI	26.9
Race	
White	98 (84%)
Black/African American	4 (3%)
Other/Unknown	15 (13%)
Rural–urban status	
Urban	99 (85%)
Rural	12 (10%)
Missing	6 (5%)
Insurance type	
Private	21 (18%)
Medicare	81 (69%)
Medicaid	2 (2%)
Missing	13 (11%)
Area deprivation index	
1–3	28 (24%)
4–10	45 (38%)
Missing	44 (38%)
Smoker	
Yes	15 (13%)
No	102 (87%)

### Tumor and treatment characteristics

Of the 119 tumors, the majority were invasive ductal carcinoma (IDC) (76%), followed by invasive lobular carcinoma (ILC) (12%), ductal carcinoma in situ (10%), and nonductal carcinomas (2%). Most of the tumors were located in the upper outer quadrant (50%) or overlapping (18%). The most common preoperative T stages were T1 (46%) or T2 (27%). Preoperatively, most patients were clinically node negative (N0) (87%) (Table 2).

**Table 2:** Tumor and treatment characteristics

	*n* (%)
*N*	119
Histologic subtype	
Invasive ductal carcinoma	91 (76%)
Invasive lobular carcinoma	14 (12%)
Ductal carcinoma in situ	12 (10%)
Other	2 (2%)
Preoperative T stage	
Tis	13 (11%)
T1	55 (46%)
T2	32 (27%)
T3	17 (14%)
T4	2 (2%)
Preoperative N stage	
N0	104 (87%)
N1	13 (11%)
N2	0 (0%)
N3	2 (2%)
Location of SLNB (laterality)	
Left	52 (44%)
Right	67 (56%)
Location of primary tumor (quadrant)	
Upper outer	59 (50%)
Lower outer	6 (5%)
Lower inner	11 (9%)
Upper inner	8 (7%)
Central	14 (12%)
Overlapping	21 (18%)
Neoadjuvant systemic therapy received	32 (27%)
Targeted dissection with previously clipped lymph node	12 (10%)
Breast surgery type	
Mastectomy	56 (47%)
Breast conserving surgery	63 (53%)
Immediate breast reconstruction	30 (25%)

Thirty-two patients received neoadjuvant systemic therapy (27%). Of the SLNBs, 56% were performed on the right axilla and 44% were performed on the left axilla. Few patients had a targeted dissection with a previously clipped lymph node (10%). Roughly equal proportions of patients underwent breast-conserving surgery (53%) and mastectomy (47%). Thirty patients underwent immediate reconstruction after mastectomy (25%) (Table 2).

### Mapping characteristics

The majority of patients mapped with at least one tracer (93.2%), while 8 patients (6.8%) did not map with either tracer. Four of the eight patients who did not map had previous ipsilateral breast surgery and radiation therapy. The other four patients who did not map had BMIs greater than 33. Twenty-six patients were found to have SLN metastases (22.2%). There was a mean and median of 1.6 and 1.0 SLNs excised per patient, respectively. The mean number of SLNs that mapped with ICG and Tc^99^ was 1.5 for both tracers. All patients who received neoadjuvant chemotherapy mapped with at least one tracer.

At the time of surgery, 199 SLNs were identified. Rates of SLN identification with ICG (89.4%) and Tc^99^ (88.4%) were similar. A majority of the SLNs mapped with both ICG and Tc^99^ (81.9%). A small portion of SLNs mapped with only ICG (7.5%) or Tc^99^ (6.5%). Eight SLNs were identified that did not map with either ICG or Tc^99^ (4.0%) and were identified by palpability or previously placed clip (Table 3). Rates of SLN identification with ICG (95.2%) were slightly better than with Tc^99^ (87.3%) for patients who received neoadjuvant chemotherapy.

**Table 3:** Sentinel lymph node mapping by tracer (*N* = 199)

	Tc+, *n* (%)	Tc−, *n* (%)	Total, *n* (%)
ICG+	163 (81.9%)	15 (7.5%)	178 (89.4%)
ICG−	13 (6.5%)	8 (4.0%)	21 (10.6%)
Total	176 (88.4%)	23 (11.6%)	199 (100.0%)

Upon pathologic examination, 230 specimens were identified. Thirty-nine SLNs were found to have metastases (17.0%); 28/39 of the positive SLNs were identified with ICG and Tc^99^, 6/39 were identified with ICG alone, 1/39 was identified with Tc^99^ alone, and 4/39 were identified by palpability or a previously placed clip. Eight of the specimens did not contain a SLN (3.5%); 4/8 of these specimens were identified with ICG and Tc^99^, 2/8 of were identified with ICG alone, and the last 2/8 were identified by palpability or a previously placed clip (Table 4). Eight of the 32 patients who received neoadjuvant chemotherapy had a cumulative 13 SLN with metastases; 7/13 positive SLNs were identified with ICG and Tc^99^, 3/13 were identified with ICG alone, 1/13 was identified with Tc^99^ alone, and 2/13 were identified by palpability or a previously placed clip.

**Table 4:** Specimens identified on pathological examination by tracer (*N* = 230)

	ICG+/Tc+ *n* (%)	ICG+/Tc− *n* (%)	ICG−/Tc+ *n* (%)	ICG−/Tc− *n* (%)	Total *n* (%)
No lymph node	4 (1.7%)	2 (0.9%)	0 (0.0%)	2 (0.9%)	8 (3.5%)
Positive	28 (12.2%)	6 (2.6%)	1 (0.4%)	4 (1.7%)	39 (17.0%)
Negative	156 (67.8%)	9 (3.9%)	15 (6.5%)	3 (1.3%)	183 (79.6%)
Total	188 (81.7%)	17 (7.3%)	16 (7.0%)	9 (3.9%)	230 (100%)

A BMI greater than 30 was associated with a significantly decreased odds of SLN detection with ICG, with an odds ratio of 0.19 (95% CI 0.04–0.99). Centrally located tumors were also associated with decreased odds of SLN detection with ICG (OR = 0.12, 95% CI: 0.02–0.78). SLN detection with ICG was not significantly associated with other patient characteristics such as age, race, or smoking status. Likewise, tumor histology and patient treatment characteristics such as surgery type, SLNB laterality, and receipt of neoadjuvant chemotherapy were also not associated with SLN detection with ICG (Table 5).

**Table 5:** Factors associated with sentinel lymph node detection by indocyanine green. Bold denotes statistical significance with p ≤ 0.05

	OR	95% CI	P value
Age at surgery (years)			
18–49	1	REF	REF
50–74	0.77	0.14–4.21	0.77
75+	0.61	0.07–5.25	0.65
Race			
White	1	REF	REF
African American	0.63	0.05–8.04	0.72
Other	1.81	0.17–19.40	0.63
BMI			
< 25	1	REF	REF
25–30	0.87	0.13–5.81	0.89
> 30	**0.19**	**0.04–0.99**	**0.05**
Smoker			
No	1	REF	REF
Yes	1.47	0.15–14.48	0.74
Histology			
Invasive Ductal Carcinoma	1	REF	REF
Invasive Lobular Carcinoma	0.30	0.05–1.66	0.17
Ductal Carcinoma in Situ	0.13	0.01–1.28	0.08
Surgery type			
Breast conserving surgery	1	REF	REF
Mastectomy	3.00	0.72–12.60	0.13
SLNB Laterality			
Right	1	REF	REF
Left	0.94	0.26–3.44	0.93
Neoadjuvant chemotherapy			
No	1	REF	REF
Yes	0.85	0.16–4.52	0.85
Tumor location			
Lateral (upper + lower outer quadrant)	1	REF	REF
Medial (upper + lower inner quadrant)	1.13	0.11–12.08	0.92
Overlap	0.91	0.09–9.28	0.94
Central	**0.12**	**0.02–0.78**	**0.03**

Bold denotes statistical significant with *p* ≤ 0.05

### Complications

Complications from SLNB were uncommon. Three patients experienced wound healing issues at the mastectomy site (2.6%). Two of these three patients experienced incisional necrosis with one requiring a subsequent irrigation and debridement and the other being managed with wound cares alone. One of the three patients experienced wound dehiscence, but had risk factors for wound dehiscence preoperatively. Only one patient experienced green breast skin discoloration on the ipsilateral side to the SLNB and this was resolved within approximately 2 weeks of the operation.

## Discussion

Staging of the axilla for patients with breast cancer is typically carried out with dual-tracer SLNB with Tc^99^ and blue dye. The aim of this retrospective study was to assess if ICG is a suitable replacement for blue dye in dual-tracer SLNB. This study demonstrated that ICG has similar SLN identification rates (89.4%) compared to Tc^99^ (88.4%) (Table 3). These results are similar to other studies that have utilized ICG in isolation or in combination with blue dye or Tc^99^ [6–17]. Of the 39 SLNs positive for metastases upon pathologic examination, most were radioactive and fluorescent (28/39, 71.8%). A notably larger number of SLNs with metastases were only fluorescent at the time of surgery (6/39, 15.4%) compared to the one SLN with metastases that was only radioactive at the time of surgery (1/39, 2.6%). This finding aligns with a previous meta-analysis that demonstrated a trend toward superior axillary staging with ICG compared to Tc^99^ for SLNB [19]. However, another meta-analysis reported no significant difference between ICG and Tc^99^ for the detection of metastatic SLNs [20].

It is suggested that neoadjuvant chemotherapy compromises SLNB, resulting in lower SLN detection rates and higher false negative rates, possibly due to compromised lymphatic drainage in the breast or irregular tumor regression in the axilla [21]. ICG is a unique tracer as it allows for real-time visualization of lymphatic channels and SLNs. One study harnessed this characteristic of ICG and demonstrated that while lymphatic drainage patterns may be altered by neoadjuvant chemotherapy, SLN detection with ICG is not [22]. Other studies have further supported the efficacy of ICG for SLNB in patients who have received neoadjuvant chemotherapy [23–25]. The current study supports the findings that ICG is a suitable tracer for patients in the neoadjuvant setting with the ICG SLN identification rate at the time of surgery being 95.2%.

There is concern that the use of ICG for SLNB may result in the removal of excess SLNs, putting patients at unnecessary risk for complications like lymphedema, nerve damage, and mobility impairments [26]. Both an earlier and more recent meta-analysis examining studies comparing ICG and blue dye for SLNB found that there were a higher number of SLNs excised with ICG compared to blue dye [27, 28]. One of these meta-analyses noted that the mean number of SLNs excised was higher for ICG (3–5.4 SLNs) than blue dye (1–2.4 SLNs) in all of the studies [27]. However, other meta-analyses comparing SLNB with ICG versus Tc^99^ demonstrated a relatively comparable number of SLNs removed with ICG (1.5–3.8 SLNs) versus Tc^99^ (1.35–2.3 SLNs) [19, 20]. A recent prospective study reported a mean of two SLNs retrieved using a combination of ICG and Tc^99^ for SLNB [9]. Another recent prospective study found similar numbers of SLNs excised among ICG alone (2.4 SLNs), ICG combined with Tc^99^ (3.1 SLNs), and ICG with blue dye (1.9 SLNs) [12]. In the current study, the mean number of SLNs detected with ICG (1.5 SLNs) was equal to Tc^99^ (1.5 SLNs) and did not reflect an excess of SLNs excised.

ICG appears to be a safe tracer for SLNB with recent studies reporting no adverse events reports such as allergic reactions, skin necrosis, or staining up to 90 days of follow-up [29, 30]. No patients in the current study experienced allergic reactions. It should be noted that absolute contraindication for ICG administration is hypersensitivity to the dye, and because the ICG dye formulation contains sodium iodide, it should be used with caution in patients with a history of iodide allergy [31]. In the current study, two patients experienced skin necrosis on the ipsilateral side of the SLNB. Both of these two patients underwent bilateral mastectomy with immediate reconstruction. It is difficult to say whether these complications are causally related to the tracer or are a result of traction injury related to the mastectomy itself or other patient factors. Lastly, one patient experienced prolonged skin discoloration after SLNB that resolved approximately two weeks postoperatively.

One described limitation of ICG is related to its detection capability which could be lower in obese patients with poor diffusion rates from lymphatic channels to the nodes, or in patients with node macrometastasis that prevent the dye from migrating through lymphatic channels to sentinel lymph nodes [32, 33]. However, successful mapping could be decreased in obese patients (BMI > 40) irrespective of the dye selection [34, 35]. A recent meta-analysis demonstrated that successful mapping with ICG was independent of patient BMI [36]. This was contradicted in the current study, where SLN detection rates with ICG were significantly decreased for BMI greater than 30. Of note, all four of the eight patients who did not map with any tracer and had no previous ipsilateral breast surgery or radiation had a BMI of 33 or greater.

Another drawback for SLNB with ICG is the necessity of SPY equipment to properly visualize fluorescent SLNs. While the upfront cost of a fluorescence imaging system can be a barrier, one study has demonstrated that even with the initial cost of an imaging system, SLNB with ICG becomes more cost-effective than SLNB with Tc^99^ within months at high-volume centers [37]. In addition, many institutions already are equipped with fluorescence imaging equipment as it is also used for assessing tissue viability, tumor locations, biliary anatomy, and so on.

There is also concern that the plastic surgeon’s ability to determine flap viability using intravenous ICG could be compromised for patients undergoing immediate reconstruction following SLNB with intradermal ICG injection. However, this can be mitigated with careful planning of intradermal injection or intratumoral injection of ICG for SLNB. In the present study, the ability of the plastic surgeons to determine tissue viability with SPY imaging and intravenous ICG was not compromised in any of the patients who underwent immediate reconstruction (25%) after SLNB with ICG.

Adopting ICG as a tracer for SLNB offers several advantages, particularly in resource-limited settings. ICG's non-radioactive nature eliminates the need for specialized facilities and regulatory approvals associated with radioactive tracers like Tc^99^, thereby reducing logistical complexities and costs. This makes SLNB more accessible in healthcare environments with limited infrastructure. Furthermore, ICG fluorescence imaging provides high sensitivity and accurate visualization of lymphatic channels and sentinel lymph nodes. One previous study has demonstrated that ICG allows for real-time visualization of lymphatic channels and sentinel lymph nodes, potentially reducing operative time and improving patient throughput, which is particularly beneficial in high-volume centers [5]. Real-time visualization with ICG also allows surgeons to quickly adjust their SLNB injection site if it is apparent that the tracer is not distributing appropriately, such as in cases where a patient has had a previous ipsilateral breast surgery or there is a bulky tumor between the nipple and axilla. Further, there is markedly less visual disruption of the tissue planes behind the nipple for patients undergoing nipple sparing mastectomy or lumpectomy for a periareolar mass with ICG compared to blue dye. These characteristics make ICG an efficient and practical alternative to traditional tracers, particularly in low-resource environments where Tc^99^ availability may be limited or restricted due to logistical or economic barriers.

This study is not without limitations. This retrospective study is susceptible to selection bias given that it was at the operating surgeon’s discretion whether or not patients had a SLNB with a combination of Tc^99^ and ICG rather than the standard combination of Tc^99^ and blue dye. This study also did not have a comparison group, and therefore, we are unable to directly compare patients who underwent SLNB with Tc^99^ and ICG versus Tc^99^ and blue dye. Lastly, this study may lack generalizability by nature of being a single institution study with two surgeons performing SLNBs.

## Conclusion

The findings of this retrospective study demonstrate that SLN identification rates using ICG and Tc^99^ are comparable to those using blue dye and Tc^99^. ICG was observed to be a safe, effective, and is a suitable alternative to blue dye in dual-tracer SLNB. However, this study found that a higher BMI was associated with a decreased likelihood of mapping with ICG. Although the number of non-mapping patients in this study was low, this is contrary to other study findings, and dye selection should be carefully considered in these patients. Nevertheless, the study’s inclusion of patients undergoing immediate reconstruction after SLNB underscores the feasibility of using ICG as a tracer for SLNB, particularly when SLNB will be followed immediate breast reconstruction using intravenous ICG for flap viability assessment. This study also uniquely included patients who had received neoadjuvant chemotherapy and provides evidence that ICG is an effective tracer for SLNB in this population. Future work should assess if ICG is suitable as a single tracer for SLNB in low-resource settings where Tc^99^ is not available or where nuclear medicine processing is not feasible.

## Data Availability

No datasets were generated or analysed during the current study.
